# Laser-Assisted High Speed Machining of 316 Stainless Steel: The Effect of Water-Soluble Sago Starch Based Cutting Fluid on Surface Roughness and Tool Wear

**DOI:** 10.3390/ma14051311

**Published:** 2021-03-09

**Authors:** Farhana Yasmin, Khairul Fikri Tamrin, Nadeem Ahmed Sheikh, Pierre Barroy, Abdullah Yassin, Amir Azam Khan, Shahrol Mohamaddan

**Affiliations:** 1 Department of Mechanical and Manufacturing Engineering, Faculty of Engineering, Universiti Malaysia Sarawak (UNIMAS), Kota Samarahan 94300, Sarawak, Malaysia; priankaipe@gmail.com (F.Y.); yabdulla@unimas.my (A.Y.); 2 Department of Mechanical Engineering, Faculty of Engineering & Technology, International Islamic University, Islamabad 44000, Pakistan; ndahmed@gmail.com; 3 Laboratoire de Physique de la Matière Condensée, Université de Picardie Jules Verne, 80025 Amiens, France; pierre.barroy@u-picardie.fr; 4 School of Chemical & Materials Engineering (SCME), National University of Sciences & Technology (NUST), Islamabad 44000, Pakistan; amir.khan@scme.nust.edu.pk; 5 Department of Bioscience and Engineering, College of System Engineering and Science, Shibaura Institute of Technology, Fukasaku 307, Minuma-ku, Saitama 337-8570, Japan; mshahrol@shibaura-it.ac.jp

**Keywords:** machining, laser-assisted milling, sago starch, surface roughness, tool wear, response surface methodology (RSM), extreme learning machine (ELM)

## Abstract

Laser-assisted high speed milling is a subtractive machining method that employs a laser to thermally soften a difficult-to-cut material’s surface in order to enhance machinability at a high material removal rate with improved surface finish and tool life. However, this machining with high speed leads to high friction between workpiece and tool, and can result in high temperatures, impairing the surface quality. Use of conventional cutting fluid may not effectively control the heat generation. Besides, vegetable-based cutting fluids are invariably a major source of food insecurity of edible oils which is traditionally used as a staple food in many countries. Thus, the primary objective of this study is to experimentally investigate the effects of water-soluble sago starch-based cutting fluid on surface roughness and tool’s flank wear using response surface methodology (RSM) while machining of 316 stainless steel. In order to observe the comparison, the experiments with same machining parameters are conducted with conventional cutting fluid. The prepared water-soluble sago starch based cutting fluid showed excellent cooling and lubricating performance. Therefore, in comparison to the machining using conventional cutting fluid, a decrease of 48.23% in surface roughness and 38.41% in flank wear were noted using presented approach. Furthermore, using the extreme learning machine (ELM), the obtained data is modeled to predict surface roughness and flank wear and showed good agreement between observations and predictions.

## 1. Introduction

A number of critical components, installed in nuclear power plants working under critical temperature ranges, are manufactured using austenitic stainless steel. This material offers high toughness, resilient strength with high ductility and thermal conductivity. Moreover, it requires high cutting force to machine; while processing it especially at higher speed and feed rate leads to excessive tool wear along with poor surface finish [1,2,3]. Nguyen, et al. [4] showed that surface roughness decreases approximately 57.65% at lower feed rate per tooth (0.09 mm/z) in dry milling of 304 stainless steel. While tool wear, which is due to adhesive wear, occurred in high speed milling of stainless steel as discussed by Liu, et al. [5].

The lowering of the mechanical strength as well as hardness of the materials at high temperature is well established. This helps in easing the machining processes especially for difficult-to-cut materials. Based on this method for the machining processes, involving thermal assistance by applying an external source of heat for softening the workpiece, can be widely effective especially for difficult-to-cut materials. Thus by employing such technique, the cutting forces as well as tool wear can be reduced significantly resulting in higher surface quality and productivity [6,7].

Lasers can be employed as an effective thermal heating source for machining processes such as high-speed milling in order to improve machinability while ensuring better surface finish and longer tool life [8,9]. Cao, et al. [10] conducted experiments on laser-assisted milling of 13-8 stainless steel to investigate surface roughness and cutting force, where results show that around 20.1% reduction in the cutting forces were observed along with 34.4% improvement in the surface roughness. Similarly using laser-assisted milling, Kim and Lee [11] observed that with the rise in the spindle speed, around 1.9 and 1.6 times reduction in the surface roughness were obtained, for AISI 1045 and Inconel 718, respectively. Recently, Attia, et al. [8] also performed an investigation on high speed laser-assisted turning of Inconel 718. A remarkable 25% improvement on the surface finish of the cut was observed while the optimal cutting speed was noted at 500 m/min. Around eight time increase of metal removal rate was observed at the optimal condition of machining. In addition, around 50% reduction of tool wear and 33% reduction in cutting forces were observed, by Bermingham, et al. [12] during laser-assisted milling of 17-4PH stainless steel. While Ito, et al. [9] demonstrated the use of the technique to machine hard and brittle fused silica at high speed. A notable 74% decrement in surface roughness was noted compared to conventional techniques but with tool life was reduced comparatively owing to direct absorption of excessive heat into the tool. In another study, up to 70% reduction in cutting forces and 50% reduction in temperature were noted by Kadivar, et al. [13] while studying laser-assisted micro-milling of austenitic stainless steel X5CrNi18-10. The results also suggest that the use of lubrication and the resultant cooling can be improved with laser-structuring.

Using the cutting fluids helps in improving tool life in addition to reducing cutting forces as well as the temperature. Those cutting fluids which have mineral-base can be hazardous to the human operators as these fluids generate toxic and foully odor fumes during the cutting. Therefore, for successful laser assisted machining, an appropriate cutting fluid formulation as well as delivery method are significant in industry [14]. As appropriate cutting fluid formulations, one way is the use of environmental-friendly cutting fluid such as vegetable-based cutting fluid, a combination of biodegradability, renewability and excellent lubrication performance [15,16]. Using vegetable-oil based nanofluids, such as coconut oil, sesame oil, and canola oil with nano molybdenum disulfide, Padmini, et al. [17] achieved better machining performance for turning substrates of AISI 1040 in terms of tool flank wear, surface roughness, cutting forces, and cutting temperatures. In the case of high speed drilling of Ti alloys, the study to understand the behaviors of different cutting fluids, it was observed by Rahim and Sasahara [18] that palm oil results in lower cutting forces and subsequent workpiece temperatures compared to the other cutting oils using synthetic ester.

However, use of vegetable oil from edible sources such as peanut oil, groundnut oil, palm kernel oil, palm oil, canola oil, and sunflower oil [17,19] will invariably trigger issues of food insecurity. They are also traditionally used as staple food in many countries. This unfortunately creates a problem for the global food security in terms of supply and price. While starch-based ingredients are widely used in food and non-food industries. Research on the application of starch as cutting fluid is very limited, reported specifically for electrical discharge machining (EDM) process using corn starch [20] and cutting of metals and non-metals using potato starch [21]. Results revealed that starch based cutting fluid has excellent cooling and lubrication properties.

In order to ensure the appropriate delivery method for cutting fluid, minimum quantity lubrication (MQL) is one good form by delivering the fluid in the mist form. This method has not only reduced the cost of machining, moreover it has led to increased machining performance [4,22,23]. It was proven as an excellent alternative to flood lubrication method by Babu, et al. [24]. In addition, results showed that the tool wear and surface roughness were respectively minimized by 70% and 66% under MQL. Using MQL with vegetable-oil cutting fluid. Bermingham, et al. [25] found five times improvement in tool life during laser-assisted milling of Ti–6Al–4V compared to conventional laser-assisted milling (dry and flood machining). Also, Khaliq, et al. [26] investigated the effectiveness of MQL in micro-milling of Ti–6Al–4V and evaluated that high cutting speed of 35,000 RPM showed improvement of tool wear under MQL condition compared to dry machining.

Predictive modelling can help assess the possible behavior of material [27,28,29] and work tool during the planning stage of machining processes and suggest suitable parametric ranges for expected better performance. Such modeling shall improve the process by reducing the losses and rejections. Moreover, the recent advancements in AI (artificial intelligence) can help by integrating with predictive models using Artificial Neural Network (ANN), genetic algorithm, and fuzzy logic. Since the data driven methods such as ANN are not only time consuming but also limited without the generalization and overfitting issues being prominent bottlenecks [30]. For example, a continual set of training data is required for ANN model to achieve generalization; however, excessive training can lead to overfitting [31]. Huang, et al. [32] proposed remedy using ELM algorithm to predict the weights of the hidden nodes. This simplification helped reduced the train-test time for ELM and provided generalization in addition to reduce the overfitting issues. Using ELM, Mustafa [33] showed that the ELM estimates are superior than ANN with better and quick cognition in lesser number of iterations. Using ELM for estimating surface roughness, Ćojbašić, et al. [34] obtained more accurate results in comparison with genetic algorithm and ANN models with smallest training error along with norm of weights. A similar observation was also made by Anicic, et al. [35]. In general, the ELM offers superiority in terms of learning speed, norm of weight, and training error [34], thus offering predictive modeling tool for controlling the parameters resulting in desired outcome.

Based on the discussion noted above, the higher productivity in higher speed milling operations can be achieved using reduction in material strength with the aid of laser assistance. To minimize environmental and food insecurity of edible oils, starch-based ingredients can be utilized as an alternative of cutting fluids or lubricants [20,21]. While, the potential of sago (Metroxylon sagu species pluralis) as an alternative source of raw material for starch is enormous. To the best of the authors’ knowledge, there is no publishable work concerning the use of sago starch as lubricating or cutting fluid in laser-assisted machining process. A study found that sago starch consists of oval granules with an average diameter of 30 µm [36]. Hence, the author anticipates that these granules may exhibit superior thermal and tribological properties similar to the nano-fluids [37,38] and potentially can be used for machining high-strength and difficult-to-cut materials.

In summary, the primary objective of this study is to compare the effect of process parameters such as spindle speed, feed rate and laser power on surface roughness of AISI316 stainless steel and tool’s flank wear width between starch based cutting fluid and conventional cutting fluid. In this study, a water-soluble sago starch based cutting fluid is prepared. Using response surface methodology (RSM) and ANOVA (Analysis of Variance), an appropriate design of experiments was used to carry out to investigate the effect of inputs on the outcomes. Furthermore, optimal input parameters are suggested to predict the surface roughness and flank wear using the models. In addition, the machined surface in terms of its integrity and the wear of flank were studied using scanning electron microscopy, optical microscope and surface roughness tester. Finally, ELM was employed to learn and predict the machining outcomes using the experimental data. The experiment is described in the following section.

## 2. Materials and Methods

### 2.1. Materials

In this study, a high strength austenitic AISI 316 stainless steel (RS PRO, Kuala Lumpur, Malaysia) of 3 mm thickness having better corrosion resistance was chosen. This alloy contains molybdenum which improves the alloy’s resistance to acids, alkalis, and chloride pitting. The chemical composition and selected mechanical and thermal properties of AISI 316 stainless steel are given in Table 1 and Table 2, respectively.

### 2.2. Experimental Details

In order to prepare the proposed water-soluble cutting fluid, sago starch was used. Since the sago starch has high viscosity, some chemical ingredients (Sigma-Aldrich, Kuala Lumpur, Malaysia) were added (shown in Table 3) to be able to use as cutting fluid. Hence, by following Fukutani, et al. [21] with little modification, cutting fluid consisting of water soluble sago-starch was obtained by dissolving carbonic acid ion and hydrogen carbonate ion in distilled water. The pH of this fluid was 10.26 at the temperature of 25 °C. Moreover, the viscosity of this water-soluble sago starch cutting fluid was measured using a viscometer (SV-10; VIBRO) (A & D Co. Ltd., Tokyo, Japan), which was 1.18 mPa.s at 25.8 °C.

**Figure 1:**
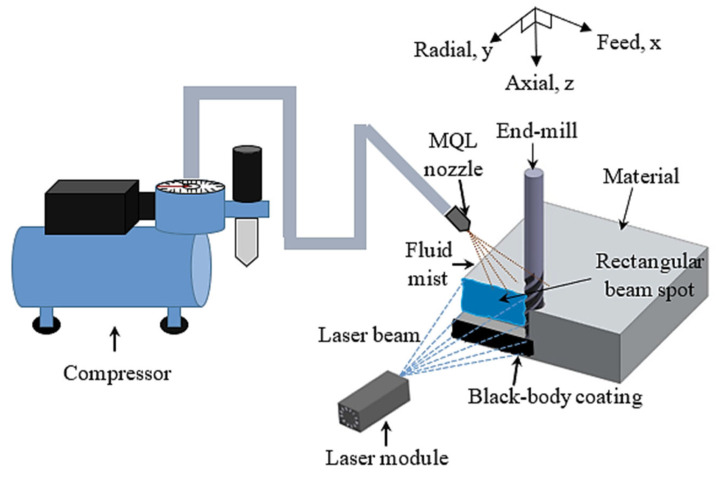
Schematic diagram of the experimental setup.

**Figure 2:**
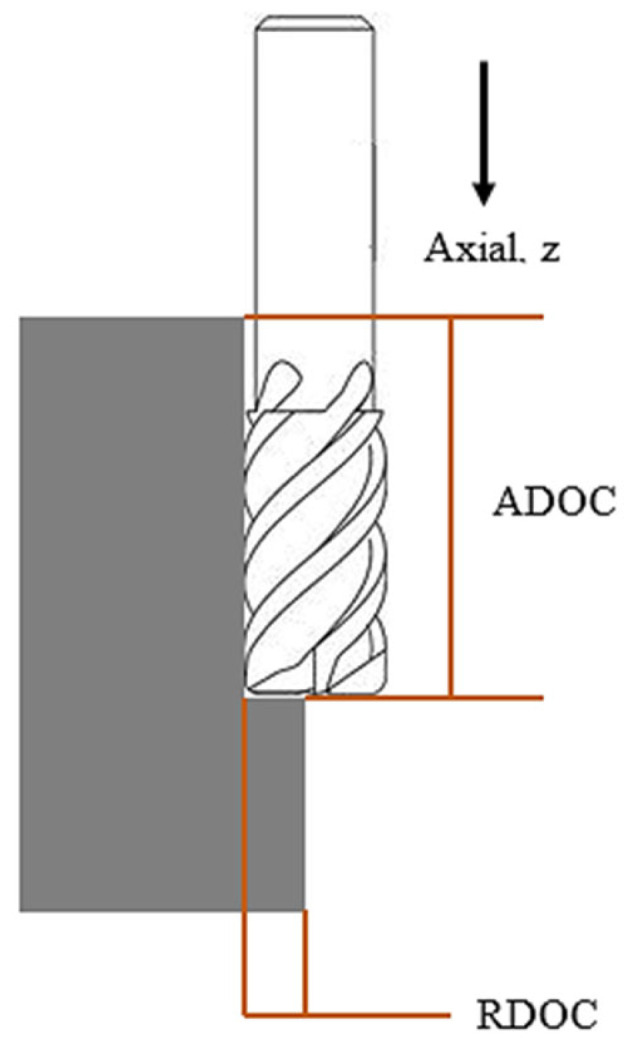
Schematic diagram of the down-cut high speed milling.

In this case, the entire experimentation was conducted on a laser-assisted high speed milling which was the combination of a cutting system, an MQL delivery system, and a continuous-heating system with laser power (Figure 1). The cutting system was down-cut milling as shown in Figure 2. For machining, X-Carve (CNC milling machine) (Inventables Inc., S. Jefferson St, Suite, Chicago) was used which has a maximum speed of 8000 mm/min with traversing rate of 500 mm/min in all three directions. The machining was carried out using an end-mill micro grain carbide coated with titanium aluminum boron nitride (AlTiBN) (WIDIN Co. Ltd., Gyeongnam), as listed Table 4. To compare the experimental results with the proposed cutting fluid, a conventional non-soluble cutting fluid was applied during machining. The dynamic viscosity of this fluids at 26.8 °C is 96.33 mPa.s. Because of the different viscosity of two fluids, the MQL flow rate of water-soluble sago starch cutting fluid and conventional cutting fluid were 5.85 mL/min and 0.33 mL/min, respectively, with 345 kPa compressed air. In addition, MQL nozzle size is 0.3 mm and the distance between the nozzle and the cutting area was 20 mm. For the entire experiments, the constant cutting parameters were shown in Table 5.

For continual heating of the stainless steel surfaces, 1.6 W diode laser (SMART DIYs, Tennessee, Japan) having the wavelength of 445 nm was employed. With the rectangular beam spot, the size of the patch matched well with the cutting area. The distance between laser module and material surface was 173 mm. A black coating on the substrate was used to increase the absorptivity of imparted energy in to the materials [41] and in addition, each substrate was pre-heated for 2 mins prior to operation.

**Figure 3:**
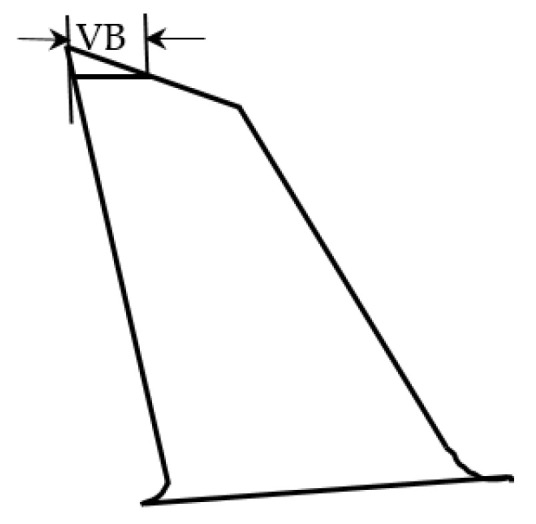
Flank wear width of end-mill based on *ISO 8688-2:1989.*

For measuring the surface roughness, SRT-6200 tester (M&A Instruments Inc., Los Angeles, CA, USA) was used, and each measurement was repeated for three times. Moreover, SEM (scanning electron microscopy) (TM3030, HITACHI, Tokyo, Japan) was employed to micrograph the machined surfaces. For the results reported here, two identical cutting tools were used for the experiments, one was for water-soluble sago starch cutting fluid and another was for conventional cutting fluid. In the case of finding the flank wear, optical microscope (MD500, AmScope, Irvine, California) having 0.57 µm/pixel resolution for 4× objectives was used following the *ISO 8688-2:1989* standard for the end-mill as shown in Figure 3.

### 2.3. Response Surface Methodology (RSM)

The systematic investigation of the processing parameters on the characteristics of the machining operation is studied using response surface methodology (RSM) as Design of Experiment (DoE). DoE helps in recovering/obtaining maximum amount of information with least number of well-planned experimentations. While RSM is used to identify the optimal configurations for the wide ranging input/process parameters for the desired outcome. Therefore, RSM was recommended in this experimental study in comparison to multiple-objective optimization [42,43,44]. While the mathematical formulation of the optimized set of parameters is obtained using regression model based on second-order polynomial equation fitted using least-squares method. The mathematic form of the model is as follows [45]:

(1) $$y=\beta_{0}+\overset{k}{\underset{i=1}{\sum }}\beta_{i}x_{i}+\overset{k}{\underset{i=1}{\sum }}\beta_{ii}x_{i}^{2}+\overset{k}{\underset{i<j}{\sum }}\beta_{ij}x_{i}x_{j}+\varepsilon$$

where *y* represents the predicted response while the machining parameters are represented by *x_i_* and *x_j_* are the value of *i*th and *j*th parameters respectively. Here, *k* is the total number of the parameters; the interaction coefficient is given by *β_i__j_*; whereas *β_i_* is the coefficient for the linear terms; The quadratic coefficient is represented by *β_ii_*; the intercept coefficient is *β_0_*, and *ε* denotes the statistical experimental error of observation. ANOVA for RSM was employed using MINITAB 18 (Minitab, LLC., Chicago) to note how the characteristics of machining are affected by the input process parameters at 95% confidence level (α = 0.05).

### 2.4. Design of Experiments

In this particular study, the spindle speed, feed rate and laser power were considered as the three major input process parameters. Prior to the final experiments, preliminary tests were performed to deduct a range of high as well as low levels of the processing parameters in order to observe the resultant surface roughness and tool wear. Since high speed milling was conducted, the spindle speed and feed rate were selected 16,000–18,200 rpm and 400–800 mm/min, respectively, based on primary tests. Above 18,200 rpm spindle speed and 800 mm/min feed rate, it was observed that the surface roughness was unacceptable, and the cutting tool absorbed much temperature. Moreover, using preliminary tests at 800 mW laser power, the levels of laser power were selected.

Response surface methodology (RSM), as a design of experiment (DoE), is a comprehensive optimization and modern mathematical statistical method [46]. RSM has been successfully employed to determine the optimal parametric combinations within a wide range of machining process parameters [45,47]. The Box-Wilson second-order central composite design (CCD) is the most commonly used in RSM since it can easily fit a second-order response surface with the minimum parameters [48]. It includes the reliable curvature estimation known for achieving a logical quantity of information in testing lack-of-fit [49,50]. In view of this, the experiments were planned using response surface methodology (RSM) based on central composite design (CCD). High and low levels of input variables were set as: High (+1), central point (0) and low (−1). Table 6 summarizes the actual as well as coded levels of the three selected variables. In full factorial design, the technique generated six axial points along with two center points and eight cube points along with four center points. In order to stabilize the estimation variance, the number of replicates runs at the center point was selected to be six. In total, 20 runs were designed as a set of experiments for both the proposed and conventional fluids.

### 2.5. Extreme Learning Machine (ELM)

Using the complete set of experimentation for learning through linear learning structure proposed by Huang, et al. [32], ELM uses the generalized inverse of Moore–Penrose for the selection of output weights with arbitrary input weights and biases. In addition, ELM is not training intensive and rather involves linear equality through one step solution, thereby quickening the process of relating input data ($x_{N}$) to output ($y_{N}$) vectors.

For the training of *i*th set, the input *x_j_* is used through a number of layers in the hidden layers to obtain output *y_j_* of the network. Using ELM, the input to output relation is defined as [32],

(2) $$y_{j}=\overset{M}{\underset{i=1}{\sum }}\beta_{i}f\left(x_{j},w_{i},b_{i}\right)$$

where $\beta_{j}$ represents the weights of the output layer and *f* designates the activation function. The weight factors (*w_j_*) relates the *j*th hidden and input nodes. While the bias of hidden nodes is represented by *b_j_*. Upon simplifications in the form of H the relationship can be expressed by Equation (4),

(3) $$H=\left[\begin{array}{ccc} f\left(x_{1},w_{1},b_{1}\right) & \ldots & f\left(x_{1},w_{M},b_{M}\right)\\ \vdots & \ldots & \vdots \\ f\left(x_{N},w_{1},b_{1}\right) & \ldots & f\left(x_{N},W_{M},b_{M}\right) \end{array}\right].$$

(4) Y=Hβ

ELM criteria is given by,

(5) $$L\left(X,Y;\beta \right)=\|Y-H\beta {\|}^{2}.$$

ELM provides an output through one step, unlike ANN [51]. Using Equation (6), *β* can be obtained using:

(6) $$\beta =H^{+}Y$$

where *H^+^* is inverse of Moore–Penrose *H* matrix.

This study uses the process parameter (inputs) to relate outputs including surface roughness and flank wear utilizing two sets of data. Out of a total of 20 data sets, random selection of 14 samples were selected for training while rest were employed to test the relationship while ensuring non-intersection. All the inputs and outputs are initially normalized on the scale −1.0 to 1.0. The analysis was carried out using MATLAB R2016a (MathWorks, Inc., Massachusetts, United States). For ELM, as the number of hidden nodes is usually selected to be less than number of training data [52]. Therefore, up to 12 hidden nodes were selected keeping in mind that 14 training sets were used. The sigmoidal function was used for activation.

## 3. Results and Discussion

Upon completion of training and testing, the resultant responses were assessed through ANOVA, to obtain the effect of input continuous variables on output and estimate the importance of each factor participating in the training. The measured characteristics of surface roughness and flank wear with water-soluble sago starch cutting fluid and conventional cutting fluid are shown in Table 7.

### 3.1. Evaluation of Model Adequacy

**Figure 4:**
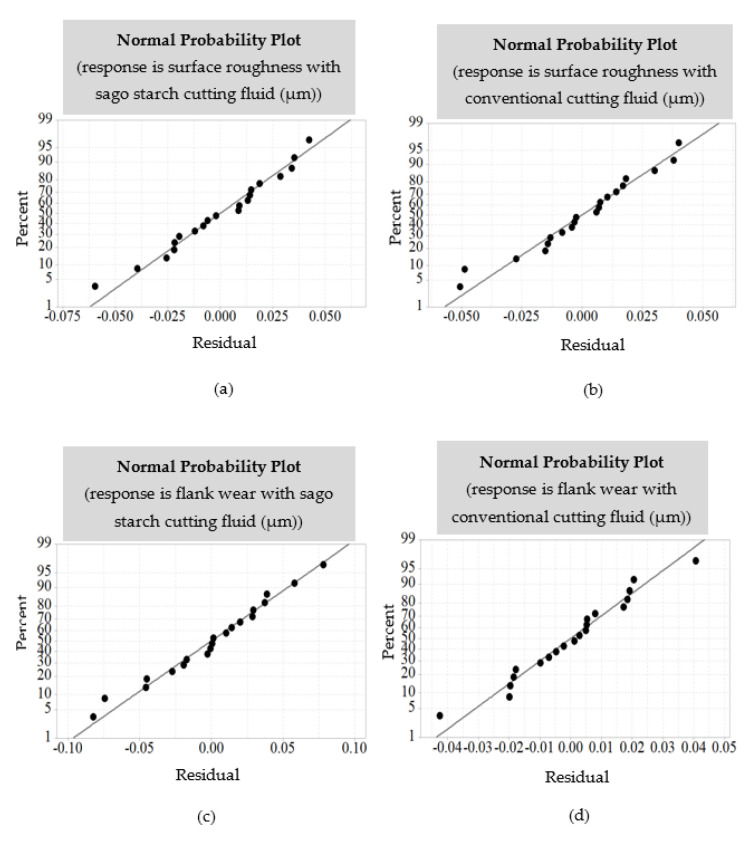
Normal probability plot of the residuals corresponding to surface roughness (**a**) with water-soluble sago starch cutting fluid, (**b**) with conventional cutting fluid, and flank wear (**c**) with water-soluble sago starch cutting fluid, and (**d**) with conventional cutting fluid.

The adequacy of the models and the normality check was carried out using the examination of residual upon fitting using regression technique. Figure 4 exhibits that the scatter of residual is well aligned in the linear plot with the normal probability plot, thus indicating that the normal assumption is properly justified.

### 3.2. Quantitative Measurement

Using RSM, a quantitative model is also proposed for the surface roughness and flank wear. The significance of the model is verified using ANOVA.

#### 3.2.1. Surface Roughness (Ra)

The ANOVA results of the surface roughness for with water-soluble sago starch cutting fluid and conventional cutting fluid are shown in Table 8 and Table 9, respectively. These reveal that the spindle speed and feed rate are the most significant parameters (*p*-value < 0.05) of surface roughness with both conditions. Though the linear power term has comparatively less effect on the roughness, this factor is also assumed as significant by 94.6% confidence level. Thus, the quadratic terms of spindle speed and feed rate has higher significance of the confidence level.

**Figure 5:**
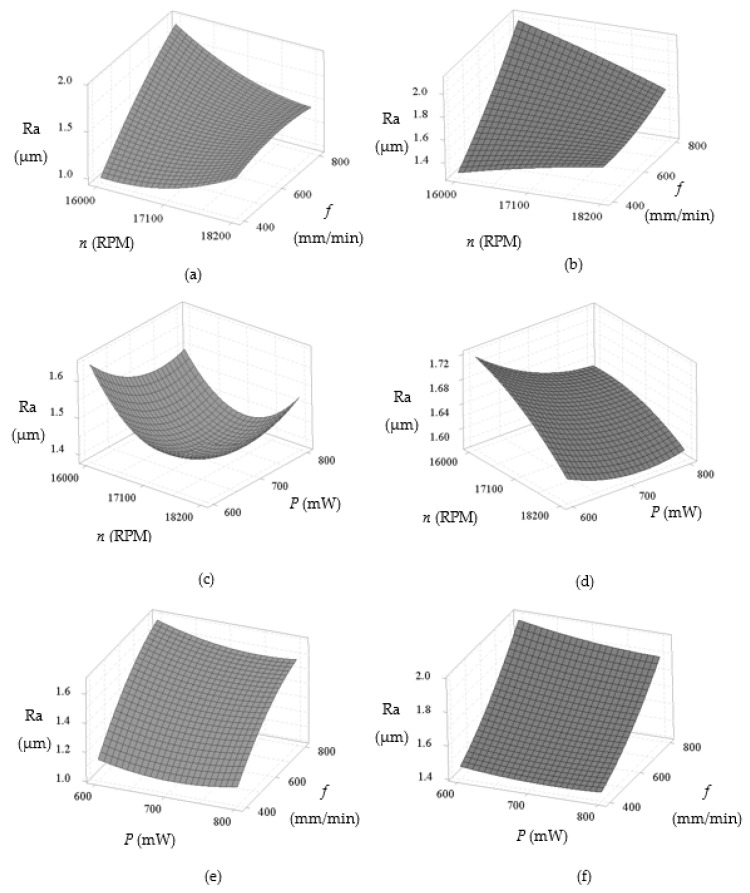
3D response surface plots of surface roughness (Ra) in terms of spindle speed—feed rate for: (**a**) Water-soluble sago starch cutting fluid and (**b**) conventional cutting fluid, spindle speed—power for: (**c**) Water-soluble sago starch cutting fluid and (**d**) conventional cutting fluid, and power—feed rate for: (**e**) Water-soluble sago starch cutting fluid and (**f**) conventional cutting fluid.

The 3D response surface plot of the surface roughness with water-soluble sago starch cutting fluid and conventional cutting fluid for different combinations of the input process parameters is shown in Figure 5, where the surface roughness is predominantly affected by spindle speed, feed rate and laser power. In both conditions, the surface roughness significantly increases with the increase of feed rate and gradually increases with the increase of spindle speed, which are shown in Figure 5a,b. However, the rate of increments of the roughness is considerably reduced with around 17,100 rpm spindle speed with the sago starch cutting fluid than conventional. By referring to Figure 5c,d, the surface finish is increased with the increase of spindle speed and power due to the reduction of cutting force with the increase of power [9,53,54]. While minimum surface roughness can be achieved at medium power and medium spindle speed with sago starch cutting fluid. In Figure 5f, the roughness linearly increases with the increment of feed rate and the decrease of laser power with conventional fluid, however, it reduces slightly with high feed rate (around 800 mm/min) with the sago starch fluid (Figure 5e). Overall, the surface finish with the proposed cutting fluid is notably improved which is maximum 48.23% compared to conventional (refer to experiment no. 16), because of the viscosity difference of the two cutting fluids. As water-soluble sago starch cutting fluid has low viscosity which is able to improve more stable lubricity [55,56]. In addition, the water-soluble cutting fluid has shown excellent lubricating properties [21].

#### 3.2.2. Flank Wear (VB)

The ANOVA results of the flank wear width with water-soluble sago starch cutting fluid and conventional cutting fluid are shown in Table 10 and Table 11. Results indicate that all input process parameters i.e., spindle speed, feed rate and laser power have most significant effect on the flank wear with both cutting fluids because of the lower *p*-value than 0.05 of the confidence level. Moreover, with the proposed cutting fluid, the two-factor interaction (2FI) terms of spindle speed and feed rate are greatly significant. While with conventional cutting fluid, only the 2FI terms of spindle speed, and the quadratic terms of spindle speed and feed rate have effect on the wear.

**Figure 6:**
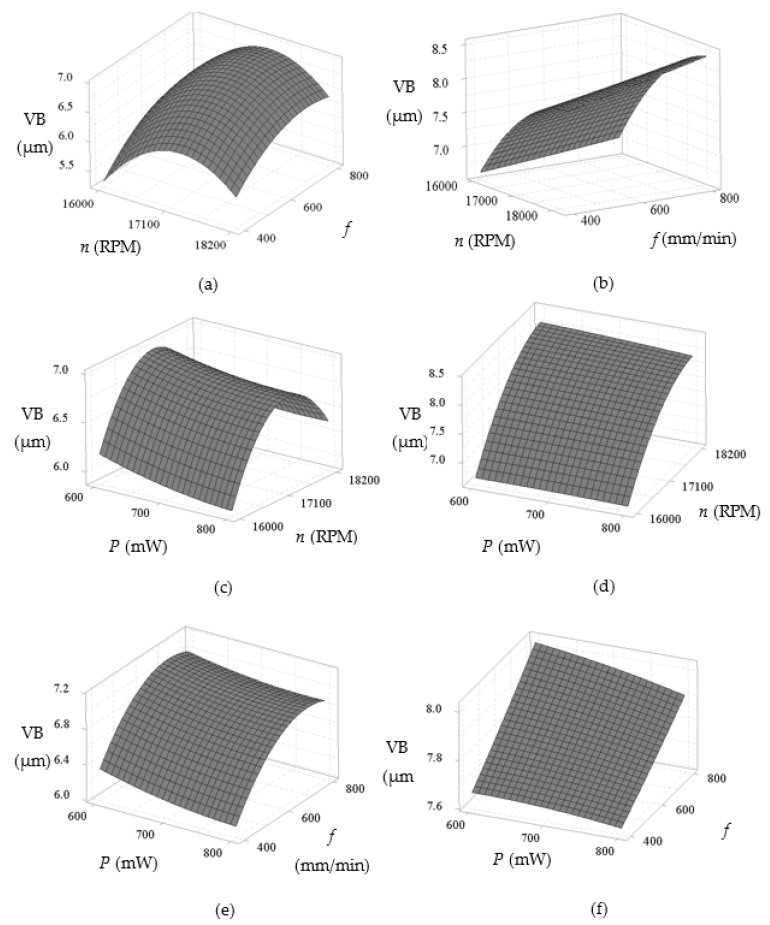
3D response surface plots of flank wear (VB) in terms of spindle speed—feed rate for: (**a**) Water-soluble sago starch cutting fluid and (**b**) conventional cutting fluid, power—spindle speed for: (**c**) Water-soluble sago starch cutting fluid and (**d**) conventional cutting fluid, and power—feed rate for: (**e**) Water-soluble sago starch cutting fluid and (**f**) conventional cutting fluid.

Figure 6 shows 3D response surface plots that reveal how the flank wear is affected by the different input process parameters with water-soluble sago starch cutting fluid and conventional cutting fluid. Based on Figure 6a,b, the flank wear increases significantly with the increase of spindle speed and gradually increases with the increase of feed rate, though the wear with conventional fluid is more pronounced at higher speed (18200 rpm) in contrast to the sago starch cutting fluid. In addition, from the Figure 6c–f, the flank wear reduces gradually for both cases, as the laser power is increased. Thus at low power, a maximum wear is obtained due to the abrasion [57]. Overall, the flank wear with water-soluble sago starch cutting fluid is considerably 38.41% reduced comparing to conventional cutting fluid (refer to experiment no. 2). This is because, water-soluble cutting fluid has excellent cooling properties which able to work with higher speed [21].

In sum, from the quantitative measurement, as spindle speed is increased, both surface roughness and flank wear reduce with the water-soluble sago starch cutting fluid (Figure 5c and Figure 6a). Thus, the spindle speed has a significant influence on these machining characteristics.

### 3.3. Qualitative Measurement

This section discusses high-speed milling operation assisted with laser source with both water-soluble sago starch cutting fluid and conventional cutting fluid under different process parameters. These experiments were performed to compare the surface morphologies of the AISI316 stainless steel substrates. The characteristics of the surface morphology and tool life using both water-soluble sago starch and conventional cutting fluids are studied.

#### 3.3.1. Surface Morphology

**Figure 7:**
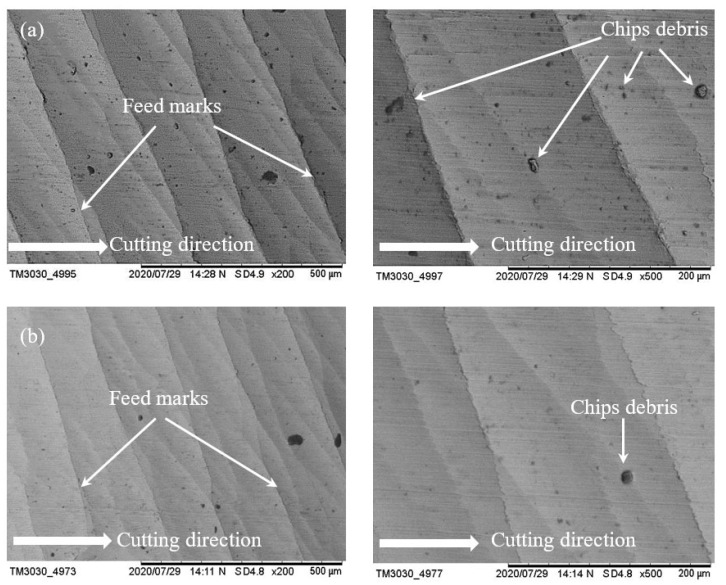
SEM micrographs of surface morphology at parametric combinations of *n* = 17,100 rpm, *f* = 400 mm/min and *P* = 700 mW; (**a**) conventional cutting fluid and (**b**) water-soluble sago starch cutting fluid.

**Figure 8:**
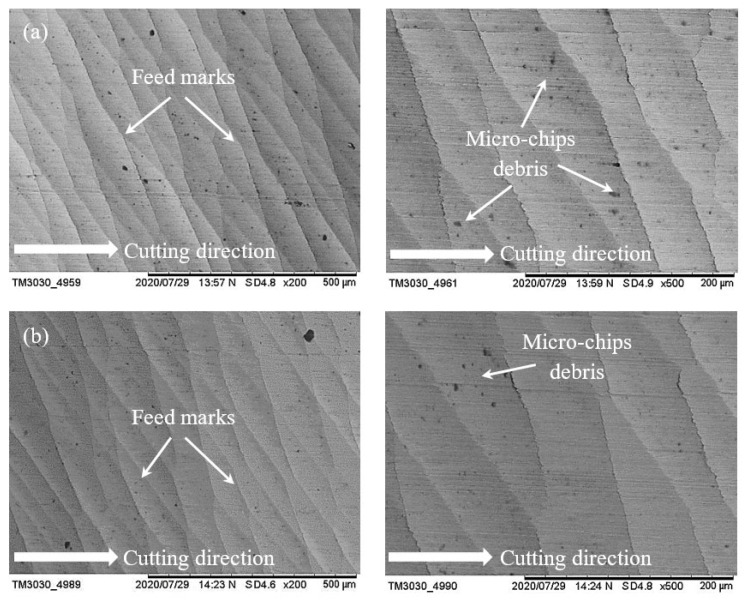
SEM micrographs of surface morphology at parametric combinations of *n* = 18,200 rpm, *f* = 400 mm/min and *P* = 600 mW; (**a**) conventional cutting fluid and (**b**) water-soluble sago starch cutting fluid.

Figure 7 shows the surface of two samples which were machined with conventional fluid and water-soluble cutting fluid at a spindle speed of 17,100 rpm, feed rate of 400 mm/min and laser power of 700 mW. For both cases, feed marks exist on the machined surface; however, the lines of feed mark are quite roughly jagged with conventional fluid (Figure 7a), while the feed mark lines are almost straight with the proposed fluid (Figure 7b). Moreover, some chips debris are adhered on the surfaces for both cases where the amount of chips debris is significantly reduced with the proposed fluid compared to conventional. As water-soluble sago starch cutting fluid has low viscosity which is able to increase the liquid flow in the pump [21] that helps to restrict the adhesion of chips. Consequently, with the proposed fluid, the finishing of machined surface is quite better than conventional. On the other hand, when the speed is increased at 18,200 rpm, it can be noticed that there is no significant difference between the machined surfaces with conventional fluid and water-soluble cutting fluid according to the feed marks which are shown in Figure 8a,b. In this case, the size of chips debris is reduced with both conditions. Nevertheless, with the sago starch fluid, the amount of micro-chips debris is less than conventional.

#### 3.3.2. Tool Life

**Figure 9:**
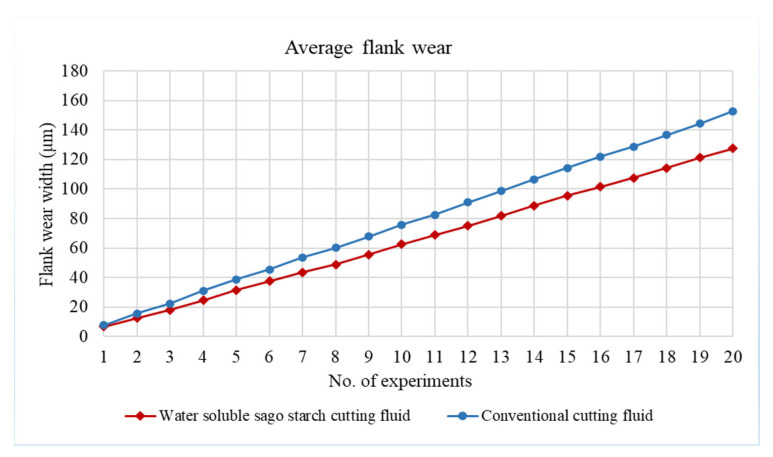
Average flank wear after conducting whole experiments.

Figure 9 shows the average flank wear width with water-soluble cutting fluid and conventional cutting fluid according to the number of experiments under the same cutting conditions. Flank wear evolution for these two cutting fluid conditions is significantly different. The tool life is observed to be around 19.64% higher with the water-soluble cutting fluid condition compared to the conventional. Moreover, flank wear is seen to be initially rapid, increasing at an approximately constant rate, which is just prior to intense wear zone [58]. While the time spent during the machining of 25 mm length stainless steel was less than five minute; the resultant relatively lesser wear width is noted in comparison to [59,60,61]. This is due to the reason that the wear reduces proportionally with the reduction of cutting time. In addition, the continual heating through laser sources also significantly minimizes the wear width. According to *ISO 8688-2:1989 Tool life testing in milling*, the end of life criterion is met when the average flank wear of end-mill reaches 0.3 mm over all teeth. In sum, after conducting total 20 runs, the average flank wears with the proposed cutting fluid and conventional are only 127.6186 µm and 152.687 µm, respectively.

**Figure 10:**
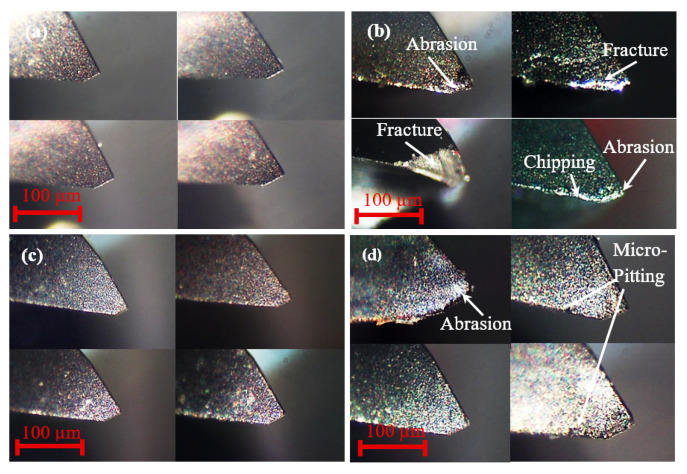
Optical micrographs of the flank wear evolution of 4-flute tool with conventional fluid (**a**) after one run, (**b**) after 20 runs, and with water-soluble sago starch cutting fluid (**c**) after one run, (**d**) after 20 runs.

Figure 10 demonstrates the different stages of the time evolution for the flank wear after one and 20 runs respectively. After machining the first experiment, there is no significant difference between the experiments with water-soluble sago starch cutting fluid and conventional cutting fluid, shown in Figure 10a,c. While after 20 runs of cutting, the wear mechanism with conventional fluid is mainly indicating a dominant fracture on the tool tip and with the signs of the abrasion on the flank face. Moreover, the machining with conventional fluid exhibits the effect of the chipping at the cutting edge which is mainly owing to the severe abrasion wear (Figure 10b). In contrast, with water-soluble sago starch cutting fluid, the flank wear occurs by normal degradation on the tool tip, i.e., abrasion and micro-pitting; however, the micro-pitting is not significant (Figure 10d). Moreover, there is no physical or apparent features such as fracture or breakages. Because, the low viscosity of the cutting fluid allows it to settle between the tool and workpiece surface/interface and provide the requisite cushion which minimizes the vibration resulting in prolong tool life [62,63].

### 3.4. Optimization

The optimal configuration of input parameters was obtained using MINITAB 18 statistical software through the response optimizer option.

#### 3.4.1. Statistical Outcome

**Figure 11:**
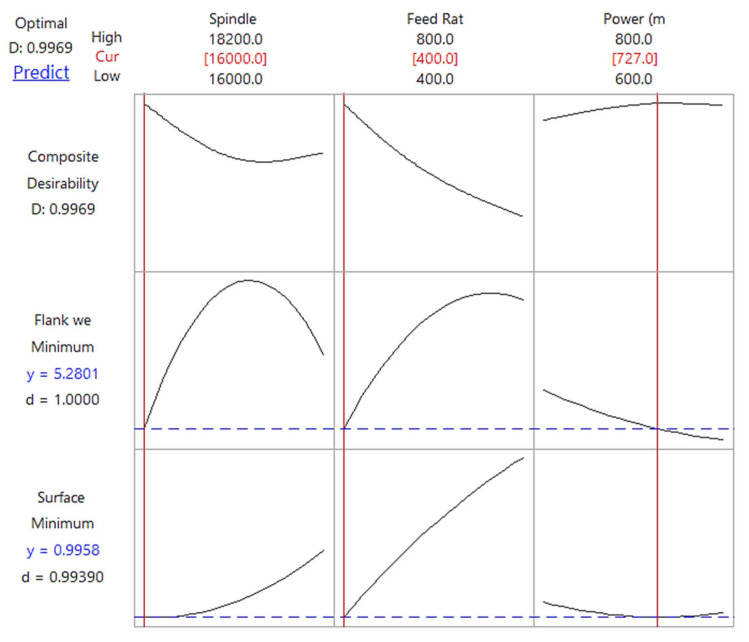
Response optimization plot of cutting parameters and the predicted results of flank wear and surface roughness with water-soluble sago starch cutting fluid.

**Figure 12:**
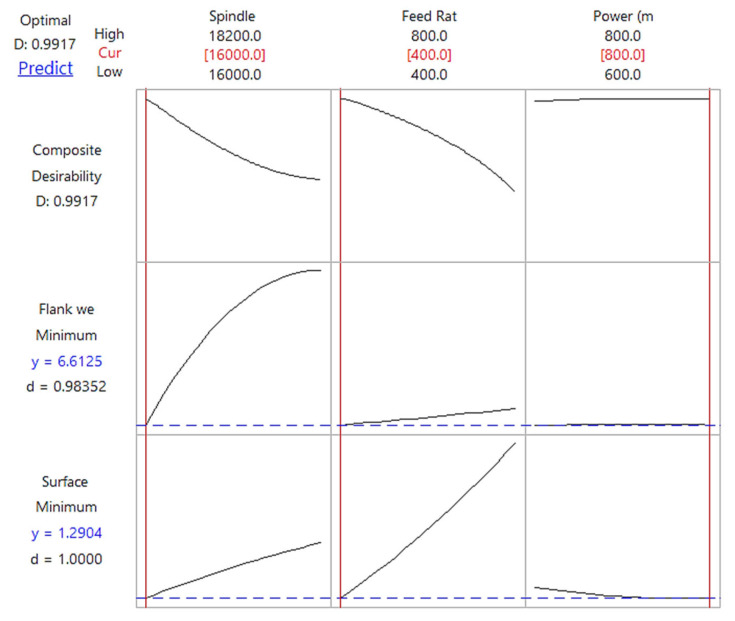
Response optimization plot of cutting parameters and the predicted results of flank wear and surface roughness with conventional cutting fluid.

The statistical outcome of optimization based on ANOVA results is shown in Figure 11 and Figure 12 with water-soluble sago starch cutting fluid and conventional cutting fluid, respectively. The predicted input parameters at a spindle speed of 16,000 rpm, feed rate of 400 mm/min and laser power of 727 mW, and the predicted values of surface roughness and flank wear are 0.9958 µm and 5.2801 µm with the proposed fluid. For the conventional fluid, at a spindle speed of 16,000 rpm, feed rate of 400 mm/min and laser power of 800 mW, the predicted surface roughness and flank wear values are 1.2904 µm and 6.6125 µm. Therefore, the notable reduction in the surface roughness and flank wear are noted to be 29.58% and 25.23%, respectively, compared to conventional cutting fluid. The accuracy of the predictions was verified using two separate runs of experiments at optimal input parametric combination. One experiment was with water-soluble sago starch cutting fluid and second experiment used conventional cutting fluid. During both of the confirmation tests, the cutting tools were new and were never used before. Table 12 compares the measured and predicted values using the regression model. The errors for both output parameters i.e., surface roughness and flank wear are 2.66% and 0.30%, respectively with the proposed cutting fluid, and 0.36% and 0.75%, respectively with conventional cutting fluid. This indicates that the experimental results are in well agreement with the predictions.

#### 3.4.2. Graphical Outcome

**Figure 13:**
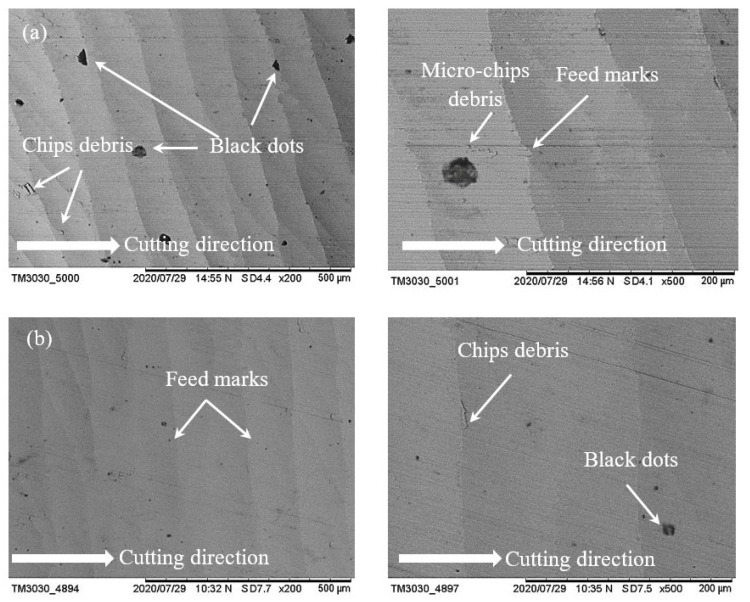
SEM photographs of machined surface at predicted parametric combinations with (**a**) conventional cutting fluid, *n* = 16,000 rpm, *f* = 400 mm/min and *P* = 800 mW, (**b**) water-soluble sago starch cutting fluid, *n* = 16,000 rpm, *f* = 400 mm/min and *P* = 727 mW.

Figure 13 shows the obtained surfaces of laser-assisted high-speed milling operation of stainless steel at optimal process parameters, where numerous chips debris with little jagged feed lines on the machined surface can be observed with conventional cutting fluid (Figure 13a). With sago starch cutting fluid (Figure 13b), it can be regarded that the presence of chips debris reduced, and the lines of feed mark are seemed straight smooth lines. Moreover, some black dots originated from the black-body coating are seen on the surface after machining with conventional fluid (Figure 13a) as compared to the water-soluble sago starch cutting fluid. This is because water-soluble cutting fluid contains some additives such as soap solution, rust-preventive agents, brighteners, and antiseptic agents which assist to remove the debris as well [21].

### 3.5. Estimation Using ELM

Using ELM, the surface roughness and flank wear are predicted both proposed and conventional cutting fluids, which are showed in Table 13 and Table 14, respectively. With water-soluble sago starch cutting fluid, the root mean square errors are minimum at 6 and 4 number hidden nodes for surface roughness and flank wear, respectively. The average errors of these machining characteristics are only 3.52% and 1.33%, respectively. With conventional cutting fluid, the number of hidden nodes is 8 and 8 to reduce the errors for surface roughness and flank wear, where the average errors are only 2.79% and 0.57%, respectively. This suggests that the observed results are in good agreement with the predictions.

## 4. Conclusions

In this present work, using RSM the effect of proposed water-soluble sago starch cutting fluid on surface roughness of AISI316 stainless steel material and tool’s flank wear during laser-assisted high speed milling were investigated. Using ELM, the observed data is compared with the predictions of the machined surface and tool condition. The following conclusions can be drawn from the obtained results.

1. The effect of single process parameter on surface roughness and flank wear were analyzed. In the levels of the parameters which were defined previously, the both surface roughness and flank wear with sago starch cutting fluid increased with an increase in the spindle speed and feed rate, and decreased with an increase in the laser power. However, the spindle speed has a significant influence on these machining characteristics. For instance, with higher spindle speed (18200 RPM), the minimum Ra and VB were 1.442 µm and 5.75 µm, respectively, with sago starch cutting fluid compared to conventional fluid (1.535 µm and 7.96 µm, respectively). Overall, with water-soluble sago starch cutting fluid, the surface roughness and flank wear reduced by 48.23% and 38.41%, respectively, compared to conventional droplet cutting fluid;
2. RSM-based optimization of the input process parameters was achieved at a spindle speed of 16,000 rpm, feed rate of 400 mm/min and laser power of 727 mW, and the predicted values of surface roughness and flank wear were 0.9958 µm and 5.2801 µm for the proposed cutting fluid. For the conventional, at a spindle speed of 16,000 rpm, feed rate of 400 mm/min and laser power of 800 mW, the predicted surface roughness and flank wear values were 1.2904 µm and 6.6125 µm. Therefore, the surface roughness and flank wear reduced by 29.58% and 25.23%, respectively, compared to conventional cutting fluid;
3. Surface morphology analysis showed that the jagged feed lines converted to the straight smooth lines and the presence of chips debris reduced with the proposed cutting fluid compared to conventional. Tool life is improved by 19.64%;
4. ELM-based prediction errors of the surface roughness and flank wear were only 3.52% and 1.33%, respectively with the proposed cutting fluid. With the conventional cutting fluid, the predicted errors of surface roughness and flank wear were only 2.79% and 0.57%, respectively, suggesting good agreement between observations and predictions.

In sum, the study is expected to be useful for laser-assisted high speed machining of various different materials due to the effect of water-soluble sago starch cutting fluid in order to improve surface finish and tool life.

However, the situation is worth stating that the internal properties of machined surface, i.e., the temperature and cutting force are critical parameters that can influence the efficiency and operational beneficial of the entire experimentation. Therefore, this calls for future investigation determining whether low-cost heat sources such as laser with high power can replace low power laser before industrialization.

## Figures and Tables

**Table 1:** Chemical composition of 316 stainless steel (wt.%) [39].

*P*	*S*	*C*	*Si*	*Mn*	*Mo*	*Ni*	*Cr*	*Fe*
≤0.002	0.01	0.074	0.35	1.06	2.22	11.61	16.92	67.75

**Table 2:** Mechanical and thermal properties of 316 stainless steel [40].

Properties	Value
Density (g/cm^3^)	8
Melting point (°C)	1370–1400
Thermal conductivity (W/m.K)	16.3
Young’s modulus (GPa)	193
Hardness Brinell (HB)	149

**Table 3:** Chemical ingredients to prepare the water-soluble sago starch cutting fluid.

Chemical Properties	Quantity
Distilled water	11 L
Sago starch	5 g
Sodium carbonate	50 g
Sodium hydrogen carbonate	30 g
Ethanol	2 mL
Dehydroacetic acid	0.5 g
Cresol and soap solution	10 mL
Rust preventive agent (linoleic acid)	10 mL

**Table 4:** Tool summary.

Parameters	Description
End-mill style	ISE1-8-4T
End-mill material	Micro-grain carbide
Coating	Titanium aluminum boron nitride (AlTiBN)
No. of flute	4
End-mill diameter	3.175 mm
Cutting length	9.525 mm
Shank diameter	3.175 mm
Full length	38.1 mm

**Table 5:** The constant cutting conditions for high speed milling machining.

Parameter	Value
Cutting length	25 mm
Radial depth of cut	0.4 mm
Axial depth of cut (depth per pass)	0.2 mm
Total depth of cut	3 mm
Plunge rate	90 mm/min

**Table 6:** Input processing parameters based on central composite design (CCD).

			Extension		
Parameter	Unit	Annotation	−1	0	+1
Spindle speed	rpm	*N*	16,000	17,100	18,200
Feed rate	mm/min	*F*	400	600	800
Laser power	mW	*P*	600	700	800

**Table 7:** Design of experiment and responses.

Experimental Input Parameter				Response			
No.	Spindle Speed (rpm)	Feed Rate (mm/min)	Power (mW)	Surface Roughness (µm)		Flank Wear (µm)	
				Water-Soluble Sago Starch Cutting Fluid	Conventional Cutting Fluid	Water-Soluble Sago starch Cutting Fluid	Conventional Cutting Fluid
1	17,100	600	700	1.408	1.638	6.8221	7.8016
2	18,200	400	800	1.442	1.535	5.7516	7.9606
3	16,000	400	600	1.097	1.334	5.5646	6.6239
4	18,200	800	600	1.497	1.737	6.4682	8.5734
5	17,100	600	700	1.412	1.635	6.8871	7.7998
6	16,000	800	800	1.912	2.077	6.0577	6.7892
7	18,200	400	600	1.464	1.556	5.9019	8.0057
8	16,000	400	800	1.079	1.310	5.2812	6.5796
9	17,100	600	700	1.403	1.630	6.8717	7.8098
10	17,100	600	700	1.399	1.632	6.8528	7.8110
11	16,000	800	600	2.100	2.225	6.2510	6.8118
12	18,200	800	800	1.477	1.709	6.4232	8.3694
13	17,100	600	800	1.387	1.611	6.6984	7.7655
14	17,100	600	600	1.432	1.659	6.9238	7.8224
15	17,100	600	700	1.405	1.640	6.8467	7.8055
16	17,100	400	700	0.989	1.466	6.0472	7.6894
17	16,000	600	700	1.474	1.598	5.8764	6.6943
18	17,100	800	700	1.582	1.856	6.8720	7.9289
19	17,100	600	700	1.412	1.636	6.8671	7.8093
20	18,200	600	700	1.464	1.604	6.3539	8.2359

**Table 8:** ANOVA results for the surface roughness with water-soluble sago starch cutting fluid.

Source	DF	Adj SS	Adj MS	*F*-Value	*p*-Value	
Spindle speed	1	0.01011	0.010112	6.00	0.040	significant
Feed rate	1	0.62350	0.623501	370.18	0.000	significant
Power	1	0.00858	0.008585	5.10	0.054	significant
Spindle speed × Spindle speed	1	0.03374	0.033736	20.03	0.002	significant
Feed rate × Feed rate	1	0.01368	0.013681	8.12	0.021	significant
Power × Power	1	0.00743	0.007429	4.41	0.069	significant
Spindle speed × Feed rate	1	0.39073	0.390728	231.98	0.000	significant
Spindle speed × Power	1	0.00336	0.003362	2.00	0.195	
Feed rate × Power	1	0.00353	0.003528	2.09	0.186	
Error	8	0.01347	0.001684			
Total	17	1.14653				

**Table 9:** ANOVA results for the surface roughness with conventional cutting fluid.

Source	DF	Adj SS	Adj MS	*F*-Value	*p*-Value	
Spindle speed	1	0.016241	0.016241	11.40	0.010	significant
Feed rate	1	0.577441	0.577441	405.34	0.000	significant
Power	1	0.007236	0.007236	5.08	0.054	significant
Spindle speed × Spindle speed	1	0.000642	0.000642	0.45	0.521	
Feed rate × Feed rate	1	0.005322	0.005322	3.74	0.089	significant
Power × Power	1	0.000922	0.000922	0.65	0.444	
Spindle speed × Feed rate	1	0.212226	0.212226	148.97	0.000	significant
Spindle speed × Power	1	0.001891	0.001891	1.33	0.283	
Feed rate × Power	1	0.002145	0.002145	1.51	0.255	
Error	8	0.011397	0.001425			
Total	17	0.845237				

**Table 10:** ANOVA results for the flank wear width with water-soluble sago starch cutting fluid.

Source	DF	Adj SS	Adj MS	*F*-Value	*p*-Value	
Spindle speed	1	0.34891	0.34891	85.58	0.000	significant
Feed rate	1	1.24299	1.24299	304.87	0.000	significant
Power	1	0.08053	0.08053	19.75	0.002	significant
Spindle speed × Spindle speed	1	1.13803	1.13803	279.13	0.000	significant
Feed rate × Feed rate	1	0.25246	0.25246	61.92	0.000	significant
Power × Power	1	0.00539	0.00539	1.32	0.283	
Spindle speed × Feed rate	1	0.00633	0.00633	1.55	0.248	
Spindle speed × Power	1	0.00990	0.00990	2.43	0.158	
Feed rate × Power	1	0.00477	0.00477	1.17	0.311	
Error	8	0.03262	0.00408			
Total	17	5.00305				

**Table 11:** ANOVA results for the flank wear width with conventional cutting fluid.

Source	DF	Adj SS	Adj MS	*F*-Value	*p*-Value	
Spindle speed	1	5.84644	5.84644	7025.29	0.000	significant
Feed rate	1	0.26034	0.26034	312.83	0.000	significant
Power	1	0.01391	0.01391	16.71	0.003	significant
Spindle speed × Spindle speed	1	0.30726	0.30726	369.21	0.000	significant
Feed rate × Feed rate	1	0.00009	0.00009	0.11	0.754	
Power × Power	1	0.00024	0.00024	0.29	0.605	
Spindle speed × Feed rate	1	0.04191	0.04191	50.35	0.000	significant
Spindle speed × Power	1	0.00415	0.00415	4.99	0.056	significant
Feed rate × Power	1	0.00235	0.00235	2.83	0.131	
Error	8	0.00666	0.00083			
Total	17	6.75597				

**Table 12:** Experimental and predicted values of surface roughness, flank wear at the optimum levels.

Method	Characteristic	Experimentation, µm	Prediction, µm	Error, %
Water-soluble sago starch cutting fluid	Surface roughness	1.023	0.9958	2.66
	Flank wear	5.2960	5.2801	0.30
Conventional cutting fluid	Surface roughness	1.295	1.2904	0.36
	Flank wear	6.5635	6.6125	0.75

**Table 13:** Comparison of extreme learning machine (ELM) prediction with experimental findings for water-soluble sago starch cutting fluid.

Run	Water-Soluble Sago Starch Cutting Fluid					
	Surface Roughness			Flank Wear		
	Experimentation (µm)	Prediction (µm)	Error %	Experimentation (µm)	Prediction (µm)	Error %
1	1.582	1.6317	3.14	6.8720	7.0973	3.28
2	1.477	1.4237	3.61	6.4232	6.4259	0.04
3	1.474	1.5446	4.79	5.8764	5.8135	1.07
4	1.464	1.4904	1.80	5.9019	5.9712	1.17
5	1.079	0.9959	7.70	5.2812	5.3600	1.49
6	1.432	1.4310	0.07	6.9238	6.9860	0.90
**Average error %**			3.52	**Average error %**		1.33

**Table 14:** Comparison of ELM prediction with experimental findings for conventional cutting fluid.

Run	Conventional Cutting Fluid					
	Surface Roughness (Ra)			Flank Wear (VB)		
	Experimentation (µm)	Prediction (µm)	Error %	Experimentation (µm)	Prediction (µm)	Error %
1	1.856	1.8449	0.60	7.9289	7.9891	0.76
2	1.709	1.6568	3.05	8.3694	8.4071	0.45
3	1.598	1.7366	8.67	6.6943	6.6961	0.03
4	1.556	1.5354	1.32	8.0057	8.0713	0.82
5	1.310	1.3023	0.59	6.5796	6.6551	1.15
6	1.659	1.7002	2.48	7.8224	7.8375	0.19
**Average error %**			2.79	**Average error %**		0.57

## Data Availability

The data presented in this study are available on request from the corresponding author.

## Nomenclature

AI	Artificial Intelligence
AlTiBN	Titanium Aluminum Boron Nitride
ANN	Artificial Neural Network
ANOVA	Analysis of Variance
CCD	Central Composite Design
DoE	Design of Experiment
EDM	Electrical Discharge Machining
ELM	Extreme Learning Machine
LAM	Laser-Assisted Machining
MQL	Minimum Quantity Lubrication
RSM	Response Surface Methodology
SEM	Scanning Electron Microscope
ADOC	Axial depth of cut
RDOC	Radial depth of cut
*n*	Spindle speed
*f*	Feed rate
*P*	Laser power
2FI	Two factor interaction
DF	Degrees of Freedom
Adj SS	Adjusted Sum of Squares
Adj MS	Adjusted Mean Squares
